# The PPARγ Agonist Rosiglitazone Suppresses Syngeneic Mouse SCC (Squamous Cell Carcinoma) Tumor Growth through an Immune-Mediated Mechanism

**DOI:** 10.3390/molecules24112192

**Published:** 2019-06-11

**Authors:** Raymond L. Konger, Ethel Derr-Yellin, Nurmukambed Ermatov, Lu Ren, Ravi P. Sahu

**Affiliations:** 1 Department of Pathology & Laboratory Medicine, Indiana University School of Medicine, Indianapolis, IN 46202, USA; ederryel@iu.edu (E.D.-Y.); Nurmukambed.Ermatov@tmcmed.org (N.E.); luren@iu.edu (L.R.); 2 Department of Dermatology, Indiana University School of Medicine, Indianapolis, IN 46202, USA; 3 Richard L. Roudebush VA Medical Center, Indianapolis, IN 46202, USA; 4 Currently in the Department of Pathology, University of Missouri-Kansas City, Kansas City, MO 64108, USA; 5 Department of Pharmacology & Toxicology, Wright State University, Dayton, OH 45435, USA; ravi.sahu@wright.edu

**Keywords:** peroxisome proliferator activated receptor gamma, rosiglitazone, T-cells, myeloid cells, anti-tumor immunity

## Abstract

Recent evidence suggests that PPARγ agonists may promote anti-tumor immunity. We show that immunogenic PDV cutaneous squamous cell carcinoma (CSCC) tumors are rejected when injected intradermally at a low cell number (1 × 10^6^) into immune competent syngeneic hosts, but not immune deficient mice. At higher cell numbers (5 × 10^6^ PDV cells), progressively growing tumors were established in 14 of 15 vehicle treated mice while treatment of mice with the PPARγ agonist rosiglitazone resulted in increased tumor rejection (5 of 14 tumors), a significant decrease in PDV tumor size, and a significant decrease in tumor cell Ki67 labeling. Rosiglitazone treatment had no effect on tumor rejection, tumor volume or PDV tumor cell proliferation in immune deficient NOD.CB17-Prkdc^SCID^/J mice. Rosiglitazone treatment also promoted an increase in tumor infiltrating CD3^+^ T-cells at both early and late time points. In contrast, rosiglitazone treatment had no significant effect on myeloid cells expressing either CD11b or Gr-1 but suppressed a late accumulation of myeloid cells expressing both CD11b and Gr-1, suggesting a potential role for CD11b^+^Gr-1^+^ myeloid cells in the late anti-tumor immune response. Overall, our data provides evidence that the PPARγ agonist rosiglitazone promotes immune-mediated anti-neoplastic activity against tumors derived from this immunogenic CSCC cell line.

## 1. Introduction

Peroxisome proliferator-activated receptor gamma (PPARγ) belongs to a subfamily of type II ligand activated nuclear receptors [1]. This subfamily includes PPARα and PPARβ. All PPAR subtypes require heterodimerization with the retinoid X receptor (RXRα) for transcriptional activity [1]. Upon ligand binding, PPAR: RXRα heterodimers promote transcriptional activity through interaction with peroxisome proliferator response elements (PPRE) of target genes [1]. Due to early observations that PPARγ was an important mediator of insulin sensitivity, thiazolidinedione (TZD) class PPARγ agonists (pioglitazone and rosiglitazone) were initially utilized as anti-diabetic agents [1].

More recently, pharmacologic evidence suggests a potential anti-cancer activity for PPARγ agonists [1,2,3,4,5,6,7,8]. Early pharmacologic studies suggested that the anti-neoplastic activity of PPARγ agonists occur through direct antitumor effects (e.g., cell cycle arrest, differentiation and cytotoxicity) [9,10,11] or through indirect mechanisms (e.g., suppression of angiogenesis) [12]. Genetic models also support an anti-neoplastic role for PPARγ: mice hemizygous for germline PPARγ exhibit an increase in spontaneous skin cancers while mice conditionally lacking epidermal PPARγ (*Pparg*-/-^epi^ mice) exhibit a marked increase in both chemical and ultraviolet B (UVB)-induced carcinogenesis [13,14,15].

In addition to potential direct effects on tumor cells, PPARγ agonists have also been shown to suppress cellular signaling pathways that are associated with the production of many inflammatory cytokines and chemokines that can recruit and activate cells of the immune system [5,16,17]. Depending on the mixture and function of the inflammatory cells that are present within the tumor microenvironment, this inflammatory environment can act in a positive manner to promote tumor growth or it can have negative effects if an anti-tumor immune response is activated [18,19]. Thus, given the known role of PPARγ in regulating inflammatory signaling, this suggests that PPARγ activation could also indirectly effect tumor growth and development through its immunomodulatory activity.

Cutaneous malignancy is primarily the consequence of highly mutagenic UVB exposure from sunlight. In addition to eliciting cancer-causing mutations, UVB can promote tumor development through the suppression of T-cell mediated anti-tumor immune responses. UVB-induced mouse skin tumors are highly immunogenic and are rejected following transplantation into syngeneic recipients [20]. In contrast, tumor rejection is suppressed if recipients are first treated with UVB to promote UVB-induced immunosuppression (UV-IS) [20]. UV-IS is also seen to suppress T-cell mediated inflammatory reactions that include contact hypersensitivity (CHS) [21]. B16F10 melanoma tumor growth is also promoted by UVB treatment (UV-IS) [21]. UVB-induced B16F10 tumor growth promotion is dependent on an intact immune system, as it is not seen in NOD.CB17-Prkdc^SCID^/J (NOD^SCID^) mice and is blocked by antibodies to interleukin (IL)-10 and the regulatory T-cell (Treg) marker CD25 [21].

The ability of UV to suppress anti-tumor immune responses as well as CHS responses is relevant to PPARγ as we have shown that *Pparg*-/-^epi^ mice in the SKH-1 hairless, albino outbred background are markedly immunosuppressed using a CHS model [1]. In addition, B16F10 melanoma tumors grow more rapidly in *Pparg*-/-^epi^ mice derived in the syngeneic C57BL/6J background [1]. Importantly, the well characterized PPARγ agonist rosiglitazone suppresses the ability of systemic UV-IS to suppress CHS responses as well as the ability of UVB treatment to promote B16F10 tumor growth [1]. This data suggests an important role for PPARγ in regulating normal and UV-induced changes in cutaneous immune responses as well as a possible role in cutaneous anti-tumor immune responses.

The idea that PPARγ can regulate immune responses is further supported by observations in other tissues. Loss of PPARγ signaling, either in the myeloid lineage itself or in lung epithelium, results in the systemic or local accumulation of immature myeloid cells with immunosuppressive activity, called myeloid-derived suppressor cells (MDSCs) [22,23]. In lung, this loss of PPARγ signaling in alveolar cells also resulted in reduced local accumulation of T-cells that resulted in an increased ratio of MDSCs relative to T-cells within the lung tissue [22].

While our earlier studies suggested that rosiglitazone could promote anti-tumor immune responses in B16F10 tumors by blocking UV-IS [1], B16F10 tumors are known to be weakly immunogenic [24]. We therefore utilized a highly immunogenic cutaneous squamous cell carcinoma (CSCC) cell line to verify a role for the well characterized PPARγ agonist rosiglitazone in promoting anti-tumor immune responses. Given the ability of PPARγ signaling to alter the balance of T-cells and myeloid cells in lung tissue, we also examined whether PPARγ altered the balance of these important immune cells in the anti-tumor response. Our data indicates that the PPARγ agonist rosiglitazone promotes anti-PDV tumor responses through an immune-mediated mechanism. Moreover, our data suggests that this may occur by regulating the local accumulation of CD3^+^ T-cells and a specific myeloid population co-expressing both CD11b and Gr-1.

## 2. Results

### 2.1. Rosiglitazone Treatment Suppresses Immunogenic PDV Tumor Growth and Promotes Tumor Regression

PDV tumor cells are a mouse CSCC cell line that were derived from 7,12-dimethylbenz[a]anthracene treated C57BL/6 mouse keratinocytes [25,26,27]. The immunogenicity of PDV tumors in syngeneic C57BL/6 mice is seen by poor tumor survival (approximately 10–20%) following subcutaneous or intradermal injection at low cell number (1 × 10^6^ cells) [25,26]. This failure to establish durable tumors has been shown to be due to T-cell mediated tumor rejection [26,28].

**Figure 1:**
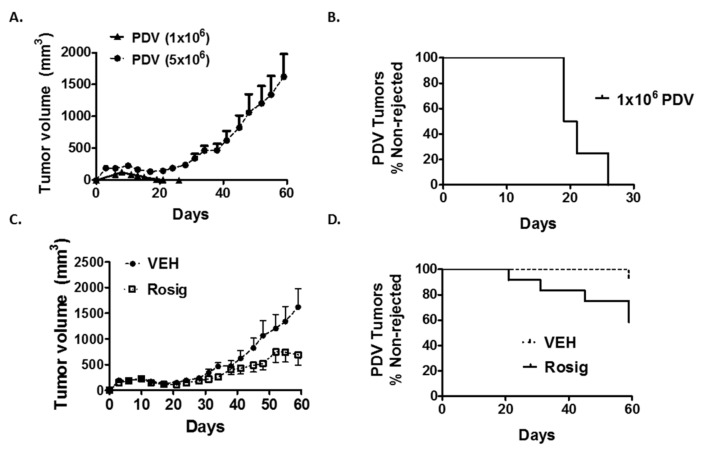
Rosiglitazone (Rosig) suppresses tumor growth and promotes tumor loss in immune competent mice. (**A**,**B**) PDV tumor survival is dependent on the number of cells that are initially injected and a biphasic tumor growth pattern is observed in durable tumors. (**A**) After the intradermal injection of 1 × 10^6^ immunogenic PDV cells into syngeneic C57BL/6J mice (*n* = 16 mice), all tumors show an initial brief increase in size followed by complete regression. When a higher number of tumor cells (5 × 10^6^) are injected (*n* = 15 mice), a two-phase tumor growth pattern occurs: An initial increase in tumor size is followed by partial regression that is then followed by a second phase of progressive tumor growth. (**B**) Immunogenic PDV tumors are reliably rejected when injected at low (1 × 10^6^) cell numbers. 1 × 10^6^ PDV tumors were injected into immune competent C57BL/6 mice. Following injection, 16 of 16 injection sites initially formed small tumors, but then the tumors began to regress in size until no visible tumors were seen. All tumors implanted with 1 × 10^6^ PDV tumor cells had completely regressed (no visible tumor) by 26 days following tumor cell injection. (**C**,**D**) Rosiglitazone (Rosig) treatment suppresses PDV tumor growth & promotes tumor rejection in immune competent mice. C57BL/6J mice were treated with 8 mg/kg/day Rosig (*n* = 14) in water or water alone (VEH) (*n* = 15) starting 10 days prior to tumor cell injection. The mice remained on Rosig or VEH for the duration of the experiment. Mice were then injected with 5 × 10^6^ PDV tumors cells and tumor size was monitored. Rosig treatment significantly reduced tumor size relative to VEH in C57BL/6J mice (*p* < 0.01 on days 21, 34, 48, 59; 2-tailed *t*-test). (**B**) Rosiglitazone treatment promotes PDV tumor rejection when tumor cells are injected at higher cell number. In contrast to PDV tumors injected at low cell numbers, 14 of 15 tumors implanted at 5 × 10^6^ cells/injection formed durable progressively growing tumors. The one rejected tumor began to regress after day 45. Upon resection at day 59, no viable tumor was observed after assessment of hematoxylin and eosin stained sections. Rosig treatment results in tumor regression that was seen as early as 21 days after tumor cell injection. In two tumors, the tumors had largely regressed by day 59, but a raised lesion was still visible. In both cases, no tumor cells were seen histologically after resection at day 59. In total, 5 of 14 tumors treated with Rosig were rejected. *p* = 0.0261, Log-rank (Mantel-Cox).

In Figure 1A,B, we verify the results of previous studies indicating that immunogenic PDV tumor cells fail to reliably establish progressively growing tumors when injected intradermally at low cell number into C57BL/6 mice. After an initial increase in tumor size during the first eight days following injection, 16/16 PDV tumors that were injected at 1 × 10^6^ cells/injection site were seen to regress and failed to form durable tumors. In Figure 1B, tumor rejection is plotted as a survival curve (% non-rejected) to illustrate how long it took for individual tumors to undergo complete regression. All tumors injected at 1 × 10^6^ cells were no longer visually apparent within 26 days.

While all tumors were rejected when 1 × 10^6^ cells were injected, when PDV tumor cells were injected at higher cell numbers (5 × 10^6^), we found that most injection sites formed progressively growing tumors in C57BL/6 mice (14/15 injection sites formed durable tumors) (Figure 1A,C,D). We also found that PDV tumors injected at higher cell numbers (5 × 10^6^) exhibited a two-phase growth curve (Figure 1A): an initial increase in tumor size that peaked around day 10–11 was followed by a partial regression in tumor size that reached its lowest point at day 17 after which we observed a resumption of progressively growing tumors.

Since immunogenic PDV tumors form durable tumors when injected at the higher cell number (5 × 10^6^), we sought to determine whether rosiglitazone treatment would alter tumor growth and tumor rejection. In Figure 1C, we show that rosiglitazone treatment results in a significant reduction in PDV tumor volume over 59 days of tumor growth when injected into C57BL/6 syngeneic hosts. This reduction in average tumor volume that was seen with rosiglitazone treatment was largely the result of an increased number of PDV tumors that rapidly regressed at some point following the initial early phase of tumor growth. The timing of tumor rejection is better illustrated in Figure 1D, which plots tumor rejection using a survival curve (% of tumors that persist and fail to undergo rejection). After 59 days of tumor growth, 5 of 14 rosiglitazone treated tumors eventually underwent complete regression during this period. Tumor rejection was spaced throughout the period of assessment, as rosiglitazone induced tumor rejection beginning as early as 21 days, but with continued tumor loss over the 59 days of observed growth. 

In several cases of late tumor regression, the tumors initially formed progressively growing tumors, but subsequently underwent a late loss in tumor volume and tumor ulceration, with no tumor visible after microscopic assessment after resection at day 59 (see example tumor growth curve in Supplemental Figure S1A). Since immunogenic PDV tumors are known to be rejected through a T-cell mediated process, we examined hematoxylin and eosin stained durable tumors excised at day 59 (see example in Supplemental Figure S1B). All tumors were surrounded by inflammatory infiltrates, while both vehicle and rosiglitazone treated tumors showed areas of lymphocytic infiltrate and on-going evidence of cytotoxic activity. This persistence of an apparent anti-tumor immune response appeared to be more extensive in surviving rosiglitazone-treated tumors, so we next verified that the ability of rosiglitazone to promote tumor regression was dependent on an intact adaptive immune system.

### 2.2. Both the Failure of PDV Tumor Cells to Establish Durable Tumors at Low Cell Numbers, as Well as the Ability of Rosiglitazone to Suppress Durable Tumor Formation, Is Dependent on an Intact Immune System

**Figure 2:**
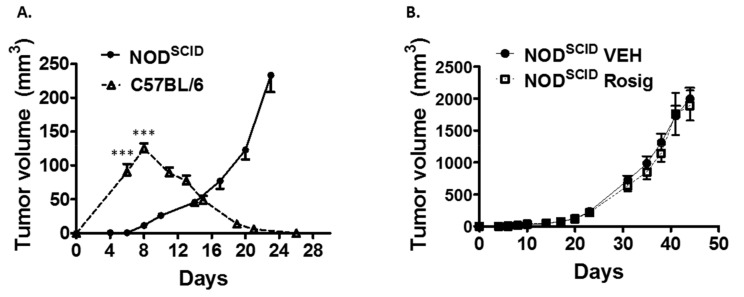
PDV tumors fail to undergo regression and rosiglitazone has no effect on tumor growth when tumor cells are injected into immune deficient mice. (**A**) The ability of PDV cells to form progressively growing tumors when injected at low cell number requires the absence of an intact adaptive immune system. PDV tumor cells were injected at low cell number (1 × 10^6^) intradermally into immune competent syngeneic C57BL/6 mice (*n* = 16) or mice lacking an intact adaptive immune system NOD.CB17-Prkdc^SCID^/J (NOD^SCID^) mice (*n* = 20). Tumor size was monitored at the indicated time points. While PDV tumors in C57BL/6 mice showed an initial rapid increase in tumor size that was followed by complete tumor regression (this data is replotted from Figure 1A), when 1 × 10^6^ PDV tumor cells were injected into immune deficient NOD^SCID^ mice, progressively growing tumors were observed, with no tumor regression. Tumor sizes were significantly different at days six and eight during the early tumor growth phase (2-tailed *t*-test). (**B**) Rosiglitazone has no effect on PDV tumor volume or rejection in *NOD^SCID^* mice. PDV tumor cells (1 × 10^6^) were injected into NOD^SCID^ mice treated with (Rosig, *n* = 18) or with water alone (VEH, *n* = 20). Rosiglitazone treatment was initiated 10 days prior to tumor cell injection as detailed in Figure 1 and the treatment was maintained throughout the tumor growth experiment.

To verify that the tumor regression seen when PDV cells are injected at low cell number is due to immune rejection, we next demonstrated that PDV cells reliably form progressively growing tumors when injected at low cell numbers (1 × 10^6^) into mice lacking functional B-cells and T-cells (NOD^SCID^ mice) (Figure 2A). Tumors injected at 1 × 10^6^ cells into immune competent hosts showed the initial increase in tumor size that peaked at day 8, followed by a complete regression of the tumors by day 26. In contrast, when 1 × 10^6^ tumor cells were injected intradermally into NOD^SCID^ mice, 20 of 20 tumors established progressively growing tumors. In addition, the rate of initial tumor size growth was much lower when PDV cells were injected into NOD^SCID^ mice. This suggests that much of the initial increase in tumor size in immune competent mice may be mediated by the inflammatory infiltrates recruited to the tumor injection site as part of the anti-tumor immune response. In Figure 2B, we show that the ability of rosiglitazone to suppress PDV tumor growth is dependent on an intact adaptive immune system. Rosiglitazone treatment had no effect on PDV tumor volume when the tumor cells are injected into NOD^SCID^ mice. In contrast to tumors in immune competent C57BL/6 mice, no tumors were seen to undergo evidence of tumor regression which strongly supports the idea that rosiglitazone treatment enhanced immune-mediated tumor rejection when the tumor cells were injected into C57BL/6 mice.

### 2.3. Immunogenic PDV Tumors Elicit an Early Inflammatory Phase That Rosiglitazone Treatment Alters by Promoting an Increase in Tumor-Infiltrating T-Cells

**Figure 3:**
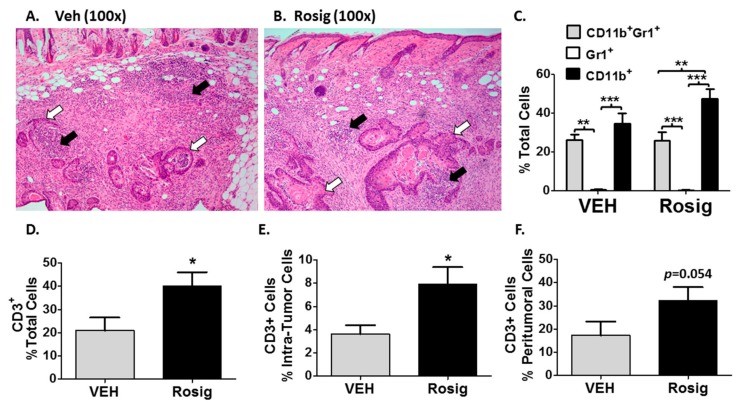
Rosiglitazone treatment promotes an increase in tumor infiltrating lymphocytes into the tumor microenvironment during the first phase of PDV tumor growth. (**A**,**B**) The first phase of tumor growth is associated with a marked inflammatory reaction. 5 × 10^6^ PDV tumor cells were injected into rosiglitazone (Rosig) or vehicle (VEH) treated C57BL/6J mice. Mice were euthanized at 11 days and formalin-fixed paraffin-embedded tumor sections were then stained with hematoxylin and eosin to assess histologic features. Tumor nests (white arrows) composed of well-differentiated SCCs were surrounded and infiltrated with a robust inflammatory cell infiltrate (black arrows). (**C**) Rosig treatment has no significant effect on the myeloid cell infiltrate seen at 11 days. In tumor sections from day 11 tumors, immunofluorescence was performed using antibodies to CD11b and Gr-1. Myeloid cells labeled with both CD11b and Gr-1, Gr-1 alone, or CD11b alone were then assessed. Rosig treatment did not result in a significant difference in the different myeloid subpopulations relative to VEH-treated controls. (*n* = 4 VEH and *n* = 6 Rosig treated tumors, 1-way ANOVA with Tukey’s Multiple Comparison’s Test). **D**–**F**.) Rosig treatment results in a significant increase in tumor infiltrating CD3^+^ cells in PDV tumors and the surrounding stroma at day 11. Immunofluorescence was performed on day 11 tumor sections using antibodies against the pan-T-cell marker CD3 and the pan-cytokeratin (pan-CK) tumor marker. Total CD3^+^ cells were assessed as a percentage of all DAPI-stained nuclei. Intra-tumoral T-cells were assessed as CD3^+^ cells adjacent to pan-CK^+^ cells and were expressed as a percentage of all pan-CK^+^ cells. Peritumoral T-cells were assessed as CD3^+^panCK^-^ cells as a percentage of total panCK^-^ cells (*n* = 5 VEH and 7 Rosig treated tumors, 2-tailed *t*-test). * *p* < 0.05; ** *p* < 0.01; *** *p* < 0.001.

We next examined whether the initial increase in tumor size seen in immune competent mice in Figure 1 and Figure 2 could represent an anti-tumor inflammatory reaction. In Figure 3A,B, photomicrographs are shown of hematoxylin and eosin stained sections obtained from tumors excised at day 11. Day 11 corresponds to the peak tumor size of this first phase of tumor growth when 5 × 10^6^ PDV cells are injected. Both vehicle and rosiglitazone treated PDV tumor nests (white arrows in Figure 3A,B) are surrounded by a dense inflammatory cell infiltrate (black arrows in Figure 3A,B). This observation is consistent with the idea that much of the tumor volume seen during this early phase of tumor growth represents largely an inflammatory reaction. In Figure 3C, the effects of rosiglitazone on myeloid cell populations was relatively minor, although rosiglitazone treatment did result in a statistically significant increase in CD11b^+^ cells relative to CD11b^+^Gr-1^+^ cells. CD11b is expressed primarily myeloid cells, such as monocytes, macrophages, eosinophils and neutrophils [29,30]. In addition, CD11b can also be expressed on CD8^+^ dendritic cells, NK cells, and a subset of CD8^+^ T cells [29]. Thus, in order to determine the significance of this finding, further work is necessary to fully characterize the CD11b^+^ cell population in each treatment group. Finally, rosiglitazone treatment failed to significantly alter the accumulation of CD11b^+^, Gr-1^+^, or CD11b^+^Gr-1^+^ myeloid cells in the tumor stroma relative to the control (VEH) mice.

In contrast to myeloid cells, when we examined the pan T-cell marker (CD3), rosiglitazone treatment resulted in a roughly 2-fold increase in total and tumor-infiltrating CD3^+^ cells (Figure 3D,E). In Figure 3F, while peritumoral CD3^+^ cells were increased in rosiglitazone treated mouse tumors, the increase fell just short of statistical significance. Combined with the inability of rosiglitazone treatment to alter PDV tumor growth in NOD^SCID^ mice, this data suggests an important role for rosiglitazone in promoting an adaptive T-cell response during the early inflammatory response to PDV tumors.

### 2.4. Rosiglitazone Alters Both the CD3 and Myeloid Cell Infiltration in PDV Tumors at Later Stages in Tumor Growth

**Figure 4:**
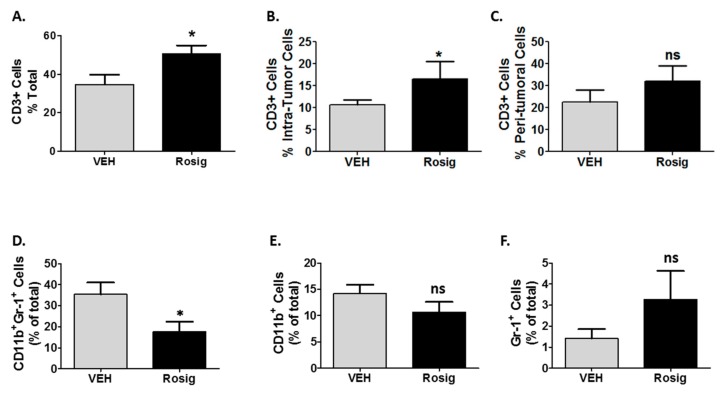
After 59 days of PDV tumor growth, rosiglitazone treatment continues to promote tumor infiltration of CD3^+^ cells but also suppresses a late increase in CD11b^+^Gr-1^+^ myeloid cells. After 59 days of tumor growth, mice were euthanized and vehicle (VEH) treated PDV tumors and the surviving rosiglitazone (Rosig)-treated PDV tumors were excised. (**A**–**C**.) Rosig treatments promotes tumor infiltrating CD3^+^ T-cells. Formalin-fixed paraffin-embedded (FFPE) tumor sections were labeled by immunofluorescence with anti-CD3 antibodies and anti-pan-CK antibodies. Tissues cytometry was then performed as detailed in Figure 3. Total infiltrating CD3^+^ T-cells, intra-tumoral CD3^+^ T-cells, and peri-tumoral CD3^+^ T-cells are shown. Rosig treatment resulted in a significant increase in CD3 cells relative to all cells in the tumor (**A**) and in intra-tumoral CD3^+^ cells (**B**) (*n* = 7 for VEH and *n* = 7 for Rosig treated groups, 2-tailed *t*-test). (**D**–**F**) PDV tumors obtained from mice treated with Rosig show a significant reduction in *CD11b^+^Gr-1^+^*myeloid cells, but no significant reduction in myeloid cells expressing only CD11b or only Gr-1. FFPE tumor sections were labeled by immunofluorescence with anti-CD11b and anti-Gr-1 as in Figure 3. Tissue cytometry was then performed to quantitate cells labeled with both CD11b and Gr-1 (CD11b^+^Gr-1^+^), CD11b alone (CD11b^+^) or Gr-1 alone (Gr-1^+^). Rosig treatment significantly decreased the numbers of myeloid cells co-expressing both CD11b and Gr-1 (**D**), but had no significant effect on myeloid cells expressing only CD11b (**E**) or Gr-1 (**F**) (*n* = 6 for VEH treated and *n* = 5 for Rosig treated groups, 2-tailed *t-*test). * p < 0.05; ns = non-significant.

While the data in Figure 3 demonstrates that an early anti-tumor inflammatory reaction occurs in response to PDV tumors, the data in Figure 1D and Figure 2B, as well as the data in Supplemental Figure S1 suggest that rosiglitazone promotes on-going immune-mediated tumor rejection that can occur at both early and late time points. To examine this ongoing anti-tumor immune response, we examined tumors excised after 59 days of growth. As with tumors excised at day 11, the data in Figure 4A–C and supplemental Figure S2 shows that rosiglitazone treatment continues to result in an increase in both total and intra-tumoral T-cell infiltrates after 59 days of tumor growth. In contrast to day 11, rosiglitazone treatment reduced CD11b^+^Gr-1^+^ cells (Figure 4D), which now represented the predominant myeloid cell population in vehicle treated mice. This differs from day 11 (Figure 3C) in which myeloid cells expressing CD11b only were the predominant myeloid cell phenotype. As with day 11, relative to vehicle treated mice, rosiglitazone treatment had no significant effect on myeloid cells expressing only CD11b^+^ or Gr-1^+^. In addition, since PDV tumors are known to be rejected through a T-cell mediated process [26,28], it is interesting that rosiglitazone treatment resulted in a late shift to a more T-cell predominant inflammatory response (CD3^+^/myeloid cell ratio > 1.0) (Supplemental Figure S3). 

### 2.5. Rosiglitazone Treatment Suppresses Cell Proliferation in PDV Tumors Grown in Immune Competent Mice, but Not in Immune Deficient Mice or in PDV Cells Grown in Cell Culture

**Figure 5:**
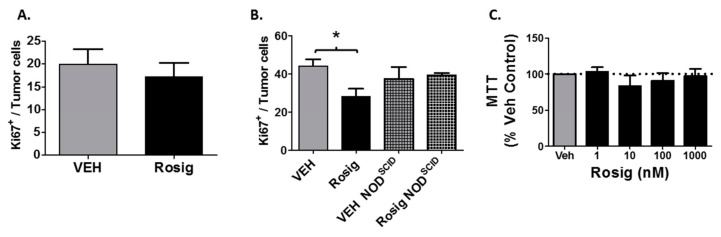
Rosiglitazone (Rosig) treatment suppresses PDV tumor cell proliferation in immune competent, but not immune deficient mice. A&B.) Rosiglitazone treatment suppresses tumor growth only in tumors after 59 days and only in immune competent hosts. Immunofluorescence staining for both Ki67 and pan-cytokeratins (pan-CK) was performed on formalin-fixed paraffin-embedded tumors. Following imaging, tumor proliferation was assessed by counting the number of Ki67^+^pan-CK^+^ double labeled cells as a percentage of total pan-CK^+^ cells. (**A**) Ki67 labeling is shown for PDV tumors that were excised at day 11 from VEH (*n* = 5) and Rosig-treated mice (*n* = 4). (**B**) Ki67 labeling is shown for PDV tumors that were excised at day 59 from C57BL/6 mice treated with VEH or Rosig (*n* = 6 each). Tumors from NOD^SCID^ mice were excised at 44 days of tumor growth (*n* = 4 each for VEH and Rosig-treated tumors). Rosig treatment significantly reduced Ki67 immunolabeling within PDV tumors at day 59 in immune competent mice (1-way ANOVA with Tukey’s Multiple Comparison test). (**C**) Rosiglitazone treatment does not alter the viability of PDV cells in culture. PDV tumor cells were grown in the presence of vehicle (Veh) or increasing concentrations of Rosig (1–1000 nM). After 48 h, cell proliferation was assessed by quantitating viable cells by MTT assay. The results represent the mean and SEM of *n* = 3 separate assays performed in quadruplicate. No significant changes were observed between any treatment group (1-way ANOVA with Tukey’s Multiple Comparison Test). * p < 0.05.

While the above data strongly suggests that rosiglitazone treatment suppresses tumor growth through its ability to stimulate anti-tumor immune responses, it is also possible that rosiglitazone could have a direct effect on PDV tumor cell proliferation. We therefore assessed the ability of rosiglitazone treatment to alter the expression of the proliferation marker Ki67 in the tumor cells. We show that rosiglitazone treatment had no significant effect on Ki67^+^ tumor cells at 11 days (Figure 5A), but significantly suppressed Ki67^+^ PDV tumor cells after 59 days in immune competent mice (Figure 5B). However, a direct effect of rosiglitazone on PDV tumor cell proliferation is unlikely, as rosiglitazone did not alter Ki67^+^ PDV tumor cell positivity when tumors were grown in immune deficient NOD^SCID^ mice (Figure 5B). In addition, rosiglitazone treatment did not cause a significant change in PDV viability after 48 h of treatment in vitro (Figure 5C). For the in vitro studies, the top concentration utilized (1000 nM) is approximately 50-fold higher than the binding affinity of rosiglitazone for PPARγ [1]. This data suggests that the ability of rosiglitazone to suppress PDV cell proliferation is indirect and dependent on an intact immune response.

## 3. Discussion

While PPARγ agonists are known to have anti-neoplastic activity, our current study indicates that the ability of the PPARγ agonist rosiglitazone to suppress the growth of immunogenic PDV tumors requires an intact adaptive immune system. While rosiglitazone suppressed tumor growth and promoted tumor rejection in immune competent syngeneic hosts, rosiglitazone had no effect on tumor growth in mice lacking functional B and T-cells. In addition, we found that rosiglitazone treatment promotes an early increase in tumor-infiltrating T-cells that preceded tumor rejection. At a later timepoint (day 59), the ability of rosiglitazone to promote CD3^+^ accumulation within the tumor microenvironment was not as pronounced. However, rosiglitazone promoted a time-dependent increase in the CD3^+^/myeloid cell ratio within the tumor microenvironment. This data is consistent with previous reports that indicated that PDV tumor rejection is dependent on the presence of T-cells [26,28]. It should be noted that 5 of 14 rosiglitazone-treated tumors and only 1 of 15 vehicle treated tumors were available for analysis at the late time point (day 59). Since the rejected tumors likely demonstrated a more robust anti-tumor immune response, it is possible that this introduced selection bias that reduced the significance of the late CD3^+^ response in the durable rosiglitazone-treated tumors. Finally, progressively growing durable PDV tumors from vehicle treated mice exhibit a late increase in CD11b^+^Gr-1^+^ myeloid cells. In contrast, rosiglitazone treatment promoted a late phase of rejection in several tumors and suppressed this late increase in this myeloid cell subpopulation. This suggests that rosiglitazone’s ability to suppress the recruitment of CD11b^+^Gr-1^+^ myeloid cells may play a role in the observed late tumor regression observed in rosiglitazone-treated mice.

Our observations share some similarities with those of another group that provides evidence that PPARγ activation may promote anti-tumor immune responses. Following immunization with a GVAX vaccine, rosiglitazone treatment promotes increased B16F10 melanoma tumor rejection, both alone and when combined with anti-CTLA4 immunotherapy [31]. As with our studies, rosiglitazone treatment resulted in an increase in tumor-infiltrating T-cells [31]. This study also suggested a potential role for myeloid cells in the rosiglitazone-mediated anti-tumor response [31]. It is also interesting that while rosiglitazone had no significant effect on weakly immunogenic B16F10 tumor growth in the absence of GVAX exposure, we show that rosiglitazone promotes highly immunogenic PDV tumor rejection in the absence of a vaccination strategy. Thus, both studies suggest that rosiglitazone can promote anti-tumor responses that are associated with both T-cell and myeloid cell changes.

Additional evidence points towards an important role for PPARγ in mediating immune responses. Studies beginning in the 1970′s by Margaret Kripke’s laboratory and verified by many others have demonstrated that ultraviolet (UV) light treatment can promote tolerance to UV-induced tumors transplanted into the UV-treated syngeneic recipients [20,21,32]. In syngeneic recipients that are not pre-treated with UV, the UV-induced tumors are rejected. UV pretreatment has also been shown to promote the growth of B16F10 melanoma tumor cells [21]. The ability of UV light to promote B16F10 tumor growth is dependent on an intact immune system, as UV light had no effect on B16F10 tumor growth in NOD^SCID^ mice [21]. UV-induced immune suppression is also seen in the ability of UV to suppress T-cell mediated contact hypersensitivity (CHS) responses. The ability of UV to suppress CHS responses as well as the ability to promote B16F10 tumor growth may be mediated through similar mechanisms that are dependent on immune tolerance-inducing signaling by interleukin-10 and CD25^+^ T-cells [21]. This association between CHS responses and anti-tumor immune responses is important as it has been previously shown that mice lacking epidermal *Pparg* (*Pparg*-/-^epi^ mice) are immune suppressed and have a severe defect in CHS responses [1]. In addition, B16F10 melanoma tumors grow more rapidly in syngeneic *Pparg*-/-^epi^ mice [1]. Finally, rosiglitazone treatment blocks the ability of UV exposure to suppress CHS responses as well as the ability of UV to promote B16F10 tumor growth [1]. Unfortunately, a clear role for the immune system in the observed responses was not definitively established.

The idea that PPARγ can regulate immune responses is also seen in other organ systems. The expression of a dominant negative *Pparg* construct (dn*Pparg*) in myeloid cells resulted in an increase in myeloid derived suppressor cell (MDSC) accumulation, increased inflammatory cytokine production, reduced CD4^+^ and CD8^+^ T-cells, and spontaneous carcinoma and sarcoma formation [23]. Unfortunately, while dn*Pparg* –expressing MDSCs were isolated and shown to suppress T-cell activation in vitro, this study failed to establish that the myeloid expression of the dn*Pparg* led to a functional defect in T-cell immunity in vivo. Similarly, it has been demonstrated that MDSC cells recruited to the lungs of mice lacking alveolar PPARγ signaling have immunosuppressive activity ex vivo [22]. A more recent study by this group demonstrates that PPARγ agonists suppress MDSC-induced tumor cell proliferation and metastatic behavior [33]. Thus, while this data from others indicates that PPARγ activation can suppress MDSC accumulation, additional work is necessary to establish that the CD11b^+^Gr-1^+^ cells observed in our studies are indeed MDSCs.

In the above studies, the authors initially assessed MDSC accumulation by assessing for myeloid cells co-expressing CD11b and Gr-1 [22,23,33]. The authors largely attributed the observed tumor formation to a proliferative microenvironment brought on by the observed chronic inflammation [33]. We show that rosiglitazone suppressed PDV tumor cell proliferation in immune competent, but not immunodeficient mice after 59 days of growth (when CD11b^+^Gr-1^+^ cells were the predominant myeloid cell type in the tumor microenvironment). However, rosiglitazone had no significant effect on tumor cell proliferation at day 11, when CD11b^+^Gr-1^+^ cells were less prominent. This data supports the idea that CD11b^+^Gr-1^+^ cells promote a local proliferative environment via a paracrine signaling mechanism. However, additional studies are needed to verify this idea. In addition, the ability of rosiglitazone to suppress CD11b^+^Gr-1^+^ cell accumulation in PDV tumors is consistent with PPARγ serving as an important inhibitor of inflammatory cytokine and chemokine production that are necessary for the recruitment of myeloid cell populations [22]. This idea is further supported by a recent study that showed that while rosiglitazone treatment had modest antitumor activity in murine CT26 colon cancer and 4T1 breast cancer tumor lines growing in BALB/c mice, a synergistic effect was seen when rosiglitazone treatment was combined with 6 or 12 Gy of radiation therapy [34]. The ability of rosiglitazone to suppress tumor growth was attributed to its ability to suppress chemokine production necessary for the recruitment of CD11b^+^ myeloid cells and tumor-associated macrophages. This supports the idea that PPARγ has important anti-inflammatory activity that acts to suppresses inflammatory cell recruitment to the skin.

Additional preclinical and clinical studies suggest a potential role for PPARγ agonists as chemotherapeutic agents, either alone or in combination with other agents. Efatutazone, a third generation TZD, combined with the anti-EGFR antibody cetuximab showed synergistic activity in suppressing tumor growth in a xenograft model [7]. In a study using monotherapy with efatutazone, 1/3 of patients with esophageal cancer achieved stable disease while 4 of 13 patients with all metastatic solid tumors achieved either stable disease or partial responses [35]. In phase I trials in various malignancies, efatutazone, either alone or in combination therapy was found to promote disease stabilization without dose-limiting toxicities [35,36,37]. Perhaps the most promising data has been obtained in oral and esophageal cancer. In a phase II human clinical trial, oral pioglitazone resulted in a partial response rate of over 70% in reducing premalignant leukoplakia lesions [2,38]. Whether these effects are dependent or independent of the immune system is unknown. However, it is clear that PPARγ agonists have been shown to have anti-neoplastic activity that is independent of an intact immune system. Efatutazone was shown to inhibit the growth of TE-4 eSCC cells xenografted into immune deficient nu/nu mice [7]. Similarly, efatutazone acted synergistically with gefitinib to suppress the growth of gefitinib-resistant lung adenocarcinoma cells in vitro [39]. Thus, PPARγ agonists may act to suppress tumor development or growth through multiple mechanisms, with direct effects on tumor growth as well as indirect effects mediated through the immune system. 

From the standpoint of direct cytotoxic effects, one dose-response study suggests that rosiglitazone can have direct tumor cytotoxicity at higher doses [40]. This group examined the effects of low (5 mg/kg) and high (20 mg/kg) doses of rosiglitazone on tumor growth in nude mice, thereby eliminating a role for the adaptive immune system in the anti-tumor response. The low dose used in this study is similar to the dose that we utilized for our studies (8 mg/kg). Importantly, they showed that while the lower doses of rosiglitazone had minimal effect on tumor growth in the immune deficient mice, the higher oral doses of rosiglitazone significantly suppressed tumor growth and promoted tumor cell apoptosis. However, it is also possible that the ability of rosiglitazone to promote direct tumor cytotoxicity is tumor dependent. As noted in Figure 5C, rosiglitazone had no effect on PDV tumor cell growth in vitro, even at concentrations well above the known binding affinity of rosiglitazone for the PPARγ receptor.

Finally, it is well known that prolonged treatment with full PPARγ agonists such as rosiglitazone are known to cause side effects such as increased weight gain and increased risk for cardiovascular events [41]. While this would not be expected to have an impact on the anti-tumor immune response to tumor growth, it does impact the potential usefulness of full PPARγ ligands as anti-neoplastic agents. However, a new class of PPARγ agonists with partial or selective PPARγ agonist activity have been shown to have anti-diabetic and anti-inflammatory effects, without the adipogenic and fluid retention side effects that are associated with full agonists like rosiglitazone [41,42,43]. Thus, future studies are needed to assess whether these partial or selective PPARγ agonists retain the anti-neoplastic activity of the full agonists that are currently in clinical use but lack the negative side effects of full PPARγ agonists such as rosiglitazone, pioglitazone or efatutazone.

In conclusion, our data supports the idea that PPARγ plays a key role in regulating immune function and that PPARγ ligands may exert anti-cancer activity through an improved anti-tumor immune response. Our data suggests that rosiglitazone treatment can alter the anti-tumor response in two ways. First, rosiglitazone promotes early tumor rejection that is associated with an increase in tumor-infiltrating T-cells. Second, rosiglitazone suppresses a second phase of tumor growth that is associated with an increase in a subset of myeloid cells co-expressing CD11b and Gr-1. This is also associated with a further increase in the ratio of CD3^+^ cells to myeloid cells populations. Thus, rosiglitazone may also suppress the ability of immunogenic tumors to initiate a delayed immune escape mechanism that may be tied to the recruitment of a specific CD11b^+^Gr-1^+^ myeloid cell population.

## 4. Materials and Methods

### 4.1. Reagents and Chemicals

Rosiglitazone maleate (rosiglitazone) was obtained from Tecoland Corp (Irvine, CA, USA).

### 4.2. Cell Line and In Vitro Proliferation Studies

PDV cells were obtained from Dr. David H. Raulet (Department of Molecular and Cell Biology, University of California, Berkeley, CA, USA) [27]. For proliferation studies, PDV cells were plated onto a 96-well plate at 1250 cells/well. The next day, the media was replaced with media containing either vehicle (ethanol) or rosiglitazone maleate at concentrations ranging from 1–1000 nM. After 48 h of growth, viable cells were quantitated by MTT (3-(4,5-dimethylthiazol-2-yl)-2,5- diphenyltetrazolium bromide) assay as previously described [1].

### 4.3. Animal Studies

C57BL/6J mice were purchased from The Jackson Laboratory (Bar Harbor, ME, USA). NOD.CB17-Prkdc^SCID^/J (NOD^SCID^) mice were purchased from the breeding colony maintained by the In Vivo Therapeutics Core within the Indiana University Melvin and Bren Simon Cancer Center. Mice were housed under specific pathogen-free conditions at the Indiana University School of Medicine. Mice utilized for experimental studies were between 7 and 10 weeks of age. The protocols were approved by the Indiana University School of Medicine Institutional Animal Care and Use Committee (IACUC, approved protocol #11256).

### 4.4. PDV Tumor Cell Injection and Rosiglitazone Treatment

Rosiglitazone treatment (40 µg/mL in water ad libitum) was initiated 10 days prior to tumor cell injection and was maintained for the duration of the tumor growth study. This dose of oral rosiglitazone (estimated at 8 mg/kg/day) was previously shown by our laboratory to significantly suppress chemical carcinogenesis in mice [41] and to block ultraviolet B-induced immune suppression [1]. Similarly, this dose is within the range of doses that have pharmacologic activity in mice [44,45]. C57BL/6J or NOD^SCID^ mice were then injected intradermally with either 1 × 10^6^ or 5 × 10^6^ PDV tumor cells. 

### 4.5. PDV Tumor Size Measurement

At the indicated time points, the tumors were measured with digital calipers in two perpendicular dimensions and the tumor volume was estimated as the length multiplied by the square of the tumor width.

### 4.6. Immunofluorescence

Immunofluorescent staining was performed on formalin-fixed paraffin-embedded (FFPE) sections. After deparaffinization, antigen retrieval was performed in R-Universal Epitope Recovery Buffer (Electron Microscopy Sciences, Hatfield, PA, USA) in a pressure cooker for 20 min. Biotin and Fc-receptor blocking was then performed. For myeloid marker assessment, sections were incubated with primary antibodies against CD11b (clone M1/70, Biotin conjugate, ThermoFisher Scientific, San Diego, CA, USA) and Gr-1 (clone RB6-8C5, FITC conjugate, ThermoFisher Scientific). For tumor-infiltrating lymphocytes, primary antibodies to the pan-T-cell marker CD3 (FLEX RTU, Dako, Agilent, Santa Clara, CA, USA) and the tumor marker (Pan-Cytokeratin (pan-CK)-AlexaFluor 488 conjugate, clone AE1/AE3, ThermoFisher Scientific) were added. For tumor cell proliferation, antibodies against Ki67 (clone SP6, ThermoFisher Scientific) and the pan-CK antibodies were utilized. All sections were incubated overnight at 4 °C. Biotinylated primary antibodies were then labeled using a secondary streptavidin-Alexa Fluor™ 594 incubation (Life Technologies Corporation, Grand Island, NY, USA). After nuclear labeling with DAPI, CD11b^+^, Gr-1^+^, and CD11b^+^Gr-1^+^ double labeled cells were assessed as a percentage of all DAPI stained nuclei. CD3^+^ labeled cells were then quantitated as peritumoral (CD3^+^CK^-^), intratumoral (CD3^+^panCK^+^) and total (CD3^+^DAPI^+^). Tumor cell proliferation was determined by counting the number of Ki67^+^panCK^+^ cells as a percentage of total panCK^+^ cells. All quantitative analysis was performed by tissue cytometry using TissueQuest software (version 3.0.1120.013720110307.01, TissueGnostics, Los Angeles, CA, USA).

### 4.7. Statistical Analysis

Group comparisons are shown as mean and standard error and were analyzed for statistical significance as detailed in each figure legend using Graphpad Prism v5.0 (Graphpad Software, Inc., San Diego, CA, USA). In the figure legends, * *p* < 0.05; ** *p* < 0.01; *** *p* < 0.001, ns = non-significant. 

## Supplementary Materials

The following are available online at https://www.mdpi.com/1420-3049/24/11/2192/s1, Figure S1: PDV tumors show evidence of on-going anti-tumor immune attack through day 59. Figure S2: Immunofluorescent staining shows an increase in tumor-infiltrating CD3^+^ cells in tumors that survived for 59 days. Figure S3: The ratio of CD3^+^ cells relative to myeloid cells within the tumor microenvironment is altered by rosiglitazone treatment.

## References

[1] Konger R.L., Derr-Yellin E., Travers J.B., Ocana J.A., Sahu R.P. (2017). Epidermal PPARγ influences subcutaneous tumor growth and acts through TNF-α to regulate contact hypersensitivity and the acute photoresponse. Oncotarget.

[2] Ondrey F. (2009). Peroxisome proliferator-activated receptor γ pathway targeting in carcinogenesis: Implications for chemoprevention. Clinical Cancer Res..

[3] Weng J.R., Chen C.Y., Pinzone J.J., Ringel M.D., Chen C.S. (2006). Beyond peroxisome proliferator-activated receptor gamma signaling: the multi-facets of the antitumor effect of thiazolidinediones. Endocr. Relat. Cancer.

[4] Yasui Y., Kim M., Tanaka T. (2008). PPAR Ligands for cancer chemoprevention. PPAR Res..

[5] Yang X.Y., Wang L.H., Farrar W.L. (2008). A Role for PPARγ in the regulation of cytokines in immune cells and cancer. PPAR Res..

[6] Zhou P., Cheng S.W., Yang R., Wang B., Liu J. (2012). Combination chemoprevention: future direction of colorectal cancer prevention. Eur. J. Cancer Prev..

[7] Sawayama H., Ishimoto T., Watanabe M., Yoshida N., Sugihara H., Kurashige J., Hirashima K., Iwatsuki M., Baba Y., Oki E. (2014). Small molecule agonists of PPAR-γ exert therapeutic effects in esophageal cancer. Cancer Res..

[8] Vella V., Nicolosi M.L., Giuliano S., Bellomo M., Belfiore A., Malaguarnera R. (2017). PPAR-γ agonists as antineoplastic agents in cancers with dysregulated IGF axis. Front. Endocrinol..

[9] Burgermeister E., Seger R. (2008). PPARγ and MEK interactions in cancer. PPAR Res..

[10] Burton J.D., Goldenberg D.M., Blumenthal R.D. (2008). Potential of peroxisome proliferator-activated receptor gamma antagonist compounds as therapeutic agents for a wide range of cancer types. PPAR Res..

[11] Hatton J.L., Yee L.D. (2008). Clinical use of PPARγ ligands in cancer. PPAR Res..

[12] Hafner C., Reichle A., Vogt T. (2005). New indications for established drugs: combined tumor-stroma-targeted cancer therapy with PPARgamma agonists, COX-2 inhibitors, mTOR antagonists and metronomic chemotherapy. Curr. Cancer Drug Targets.

[13] Nicol C.J., Yoon M., Ward J.M., Yamashita M., Fukamachi K., Peters J.M., Gonzalez F.J. (2004). PPARgamma influences susceptibility to DMBA-induced mammary, ovarian and skin carcinogenesis. Carcinogenesis.

[14] Indra A.K., Castaneda E., Antal M.C., Jiang M., Messaddeq N., Meng X., Loehr C.V., Gariglio P., Kato S., Wahli W. (2007). Malignant transformation of DMBA/TPA-induced papillomas and nevi in the skin of mice selectively lacking retinoid-X-receptor alpha in epidermal keratinocytes. J. Invest. Dermatol..

[15] Sahu R.P., DaSilva S.C., Rashid B., Martel K.C., Jernigan D., Mehta S.R., Mohamed D.R., Rezania S., Bradish J.R., Armstrong A.B. (2012). Mice lacking epidermal PPARγ exhibit a marked augmentation in photocarcinogenesis associated with increased UVB-induced apoptosis, inflammation and barrier dysfunction. Int. J. Cancer.

[16] Glass C.K., Saijo K. (2010). Nuclear receptor transrepression pathways that regulate inflammation in macrophages and T cells. Nature Rev. Immunol..

[17] Ricote M., Glass C.K. (2007). PPARs and molecular mechanisms of transrepression. BBA Mol. Cell Biol. Lipids.

[18] Thorsson V., Gibbs D.L., Brown S.D., Wolf D., Bortone D.S., Ou Yang T.-H., Porta-Pardo E., Gao G.F., Plaisier C.L., Eddy J.A. (2018). The immune landscape of cancer. Immunity.

[19] Hanahan D., Weinberg R.A. (2011). Hallmarks of cancer: The next generation. Cell.

[20] Kripke M.L. (2013). Reflections on the field of photoimmunology. J. Invest. Dermatol..

[21] Sahu R.P., Turner M.J., DaSilva S.C., Rashid B.M., Ocana J.A., Perkins S.M., Konger R.L., Touloukian C.E., Kaplan M.H., Travers J.B. (2012). The environmental stressor ultraviolet B radiation inhibits murine antitumor immunity through its ability to generate platelet-activating factor agonists. Carcinogenesis.

[22] Wu L., Wang G., Qu P., Yan C., Du H. (2011). Overexpression of dominant negative peroxisome proliferator-activated receptor-γ (PPARγ) in alveolar type II epithelial cells causes inflammation and T-Cell suppression in the lung. Am. J. Pathol..

[23] Wu L., Yan C., Czader M., Foreman O., Blum J.S., Kapur R., Du H. (2012). Inhibition of peroxisome proliferator-activated receptor-γ in myeloid lineage cells induces systemic inflammation, immunosuppression and tumorigenesis. Blood.

[24] Ngiow S.F., von Scheidt B., Akiba H., Yagita H., Teng M.W.L., Smyth M.J. (2011). Anti-TIM3 antibody promotes T Cell IFN-γ–mediated antitumor immunity and suppresses established tumors. Cancer Res..

[25] Caulin C., Bauluz C., Gandarillas A., Cano A., Quintanilla M. (1993). Changes in keratin expression during malignant progression of transformed mouse epidermal keratinocytes. Exp. Cell Res..

[26] Girardi M., Oppenheim D., Glusac E.J., Filler R., Balmain A., Tigelaar R.E., Hayday A.C. (2004). Characterizing the protective component of the αβ T cell response to transplantable squamous cell carcinoma. J. Invest. Dermatol..

[27] Whang M.I., Guerra N., Raulet D.H. (2009). Costimulation of dendritic epidermal γδ T Cells by a new NKG2D ligand expressed specifically in the skin. J. Immunol..

[28] Girardi M., Oppenheim D.E., Steele C.R., Lewis J.M., Glusac E., Filler R., Hobby P., Sutton B., Tigelaar R.E., Hayday A.C. (2001). Regulation of cutaneous malignancy by γδ T cells. Science.

[29] Christensen J.E., Andreasen S.Ø., Christensen J.P., Thomsen A.R. (2001). CD11b expression as a marker to distinguish between recently activated effector CD8+ T cells and memory cells. Int. Immunol..

[30] Kanwar S., Smith C.W., Shardonofsky F.R., Burns A.R. (2001). The role of Mac-1 (CD11b/CD18) in antigen-induced airway eosinophilia in mice. Am. J. Respir. Cell Mol. Biol..

[31] Goyal G., Wong K., Nirschl C.J., Souders N., Neuberg D., Anandasabapathy N., Dranoff G. (2018). PPAR-γ contributes to immunity by cancer vaccines that secrete GM-CSF. Cancer Immunol. Res..

[32] De Gruijl F.R. (2008). UV-induced immunosuppression in the balance†. Photochem. Photobiol..

[33] Zhao T., Du H., Blum J.S., Yan C. (2016). Critical role of PPARγ in myeloid-derived suppressor cell-stimulated cancer cell proliferation and metastasis. Oncotarget.

[34] Huang G., Yin L., Lan J., Tong R., Li M., Na F., Mo X., Chen C., Xue J., Lu Y. (2018). Synergy between peroxisome proliferator-activated receptor gamma agonist and radiotherapy in cancer. Cancer Sci..

[35] Murakami H., Ono A., Takahashi T., Onozawa Y., Tsushima T., Yamazaki K., Jikoh T., Boku N., Yamamoto N. (2014). Phase I study of Efatutazone, an oral PPARgamma agonist, in patients with metastatic solid tumors. Anticancer Res..

[36] Komatsu Y., Yoshino T., Yamazaki K., Yuki S., Machida N., Sasaki T., Hyodo I., Yachi Y., Onuma H., Ohtsu A. (2014). Phase 1 study of efatutazone, a novel oral peroxisome proliferator-activated receptor gamma agonist, in combination with FOLFIRI as second-line therapy in patients with metastatic colorectal cancer. Invest. New Drugs.

[37] Pishvaian M.J., Marshall J.L., Wagner A.J., Hwang J.J., Malik S., Cotarla I., Deeken J.F., He A.R., Daniel H., Halim A.B. (2012). A phase 1 study of efatutazone, an oral peroxisome proliferator-activated receptor gamma agonist, administered to patients with advanced malignancies. Cancer.

[38] Ondrey F.G. Pioglitazone in oral leukoplakia: a phase II trial. Proceedings of the American Association for Cancer Research International Conference: Frontiers in Cancer Prevention Research.

[39] Ni J., Zhou L.L., Ding L., Zhao X., Cao H., Fan F., Li H., Lou R., Du Y., Dong S. (2017). PPARgamma agonist efatutazone and gefitinib synergistically inhibit the proliferation of EGFR-TKI-resistant lung adenocarcinoma cells via the PPARgamma/PTEN/Akt pathway. Exp. Cell Res..

[40] Girnun G.D., Naseri E., Vafai S.B., Qu L., Szwaya J.D., Bronson R., Alberta J.A., Spiegelman B.M. (2007). Synergy between PPARγ ligands and platinum-based drugs in cancer. Cancer Cell.

[41] Ren L., Konger R.L. (2019). Evidence that peroxisome proliferator-activated receptor γ suppresses squamous carcinogenesis through anti-inflammatory signaling and regulation of the immune response. Mol. Carcinogen..

[42] Liu K., Black R.M., Acton Iii J.J., Mosley R., Debenham S., Abola R., Yang M., Tschirret-Guth R., Colwell L., Liu C. (2005). Selective PPARγ modulators with improved pharmacological profiles. Bioorg. Med. Chem. Lett..

[43] Tan Y., Muise E.S., Dai H., Raubertas R., Wong K.K., Thompson G.M., Wood H.B., Meinke P.T., Lum P.Y., Thompson J.R. (2012). Novel transcriptome profiling analyses demonstrate that selective PPARγ modulators display attenuated and selective gene regulatory activity in comparison with PPARγ full agonists. Mol. Pharm..

[44] Hwang J., Kleinhenz D.J., Rupnow H.L., Campbell A.G., Thulé P.M., Sutliff R.L., Hart C.M. (2007). The PPARγ ligand, rosiglitazone, reduces vascular oxidative stress and NADPH oxidase expression in diabetic mice. Vascul. Pharmacol..

[45] Hindlet P., Barraud C., Boschat L., Farinotti R., Bado A., Buyse M. (2012). Rosiglitazone and metformin have opposite effects on intestinal absorption of oligopeptides via the proton-dependent PepT1 transporter. Mol. Pharm..

