# Supplementary material for: The PPARγ Agonist Rosiglitazone Suppresses Syngeneic Mouse SCC (Squamous Cell Carcinoma) Tumor Growth through an Immune-Mediated Mechanism
Source: Molecules. 2019 Jun 11;24(11):2192. doi: 10.3390/molecules24112192 (PMC6600265; doi:10.3390/molecules24112192)

Supplementary Information: Konger, R.L. *et al.* The PPARγ agonist rosiglitazone suppresses syngeneic mouse SCC tumor growth through an immune-mediated mechanism.

A.

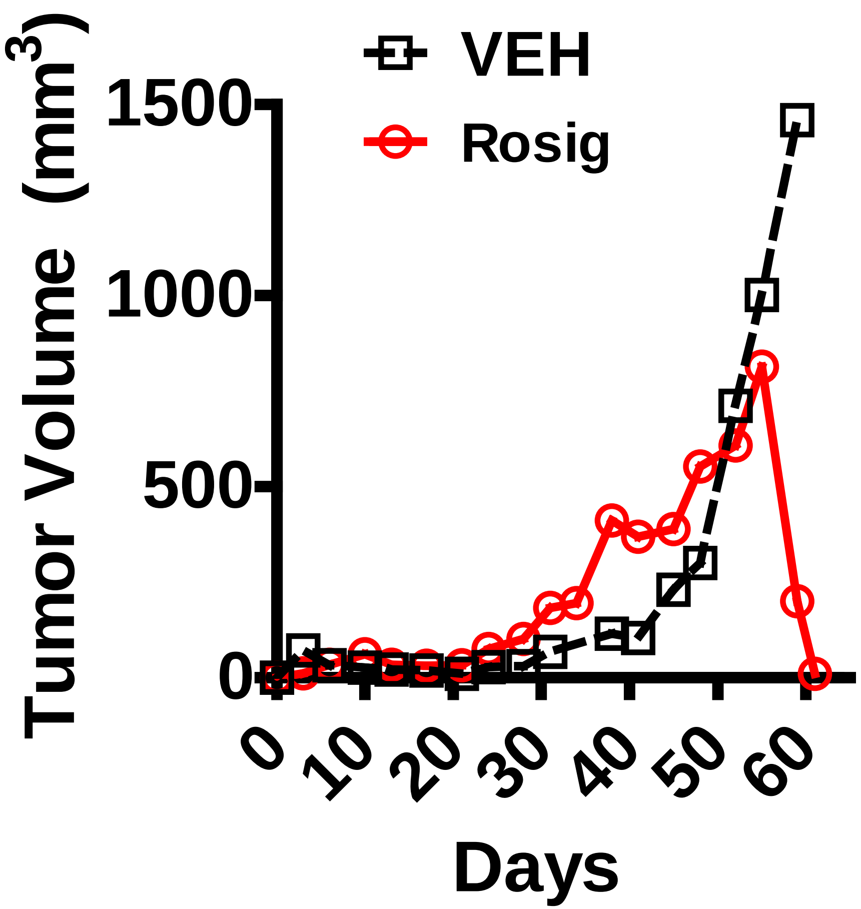

B.

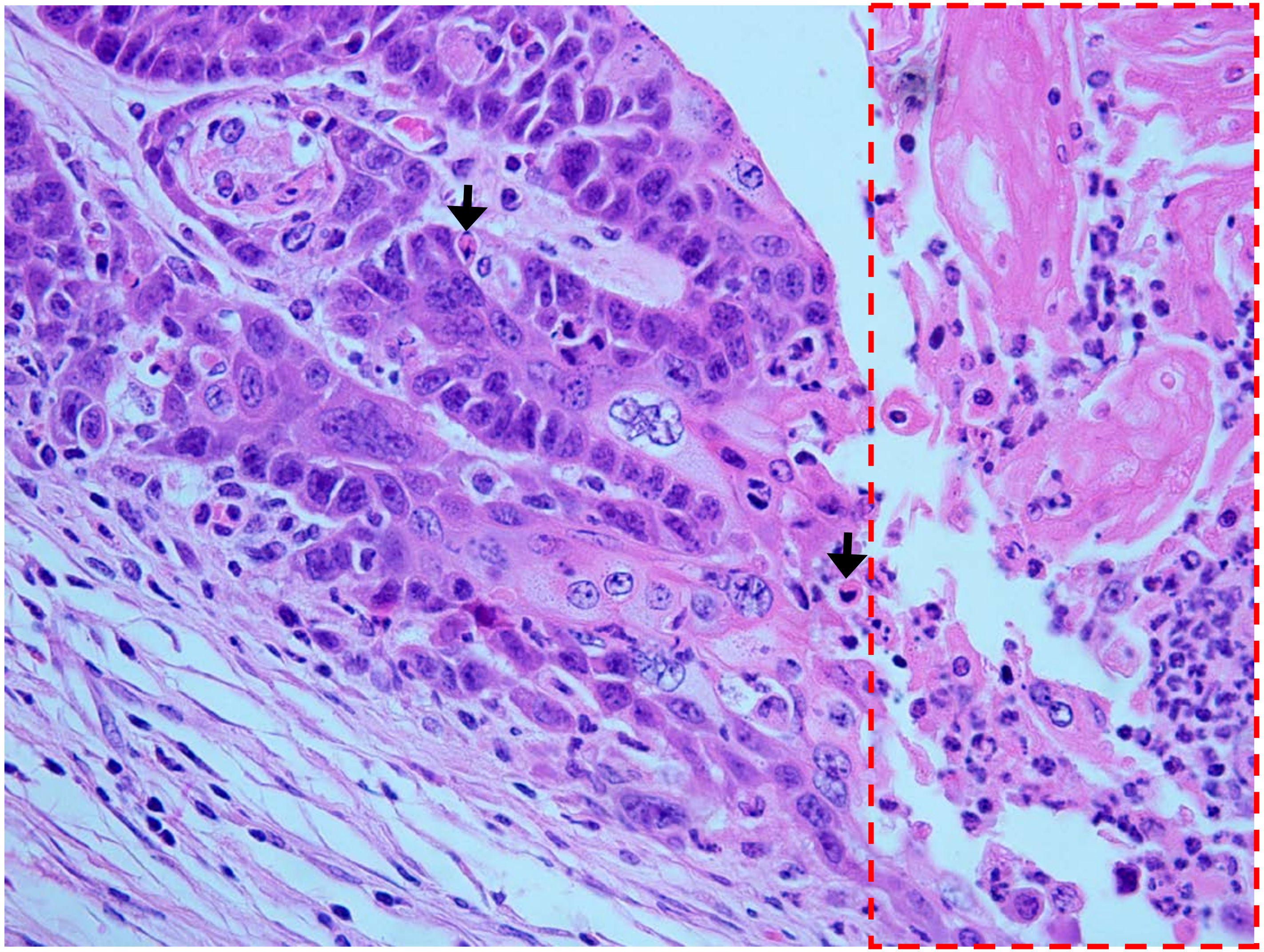

Figure S1: PDV tumors show evidence of on-going anti-tumor immune attack through day 59. A.) Delayed tumor rejection is shown for a rosiglitazone (Rosig)-treated tumor. The tumor showed progressive increases in tumor size for over 50 days, followed by an abrupt decrease in tumor size. No visible viable tumor was seen at 59 days by H&E stain. A vehicle treated tumor with progressive tumor growth is also shown. **B.)** H&E stained photomicrograph (400x) of a rosiglitazone treated tumor excised at day 59. Areas of central necrosis & cornification with neutrophilic infiltrates were noted (area inside red hash rectangle). This feature was observed in both rosiglitazone and vehicle controls. Tumors also showed numerous infiltrating lymphocytes and apoptotic bodies (arrows).

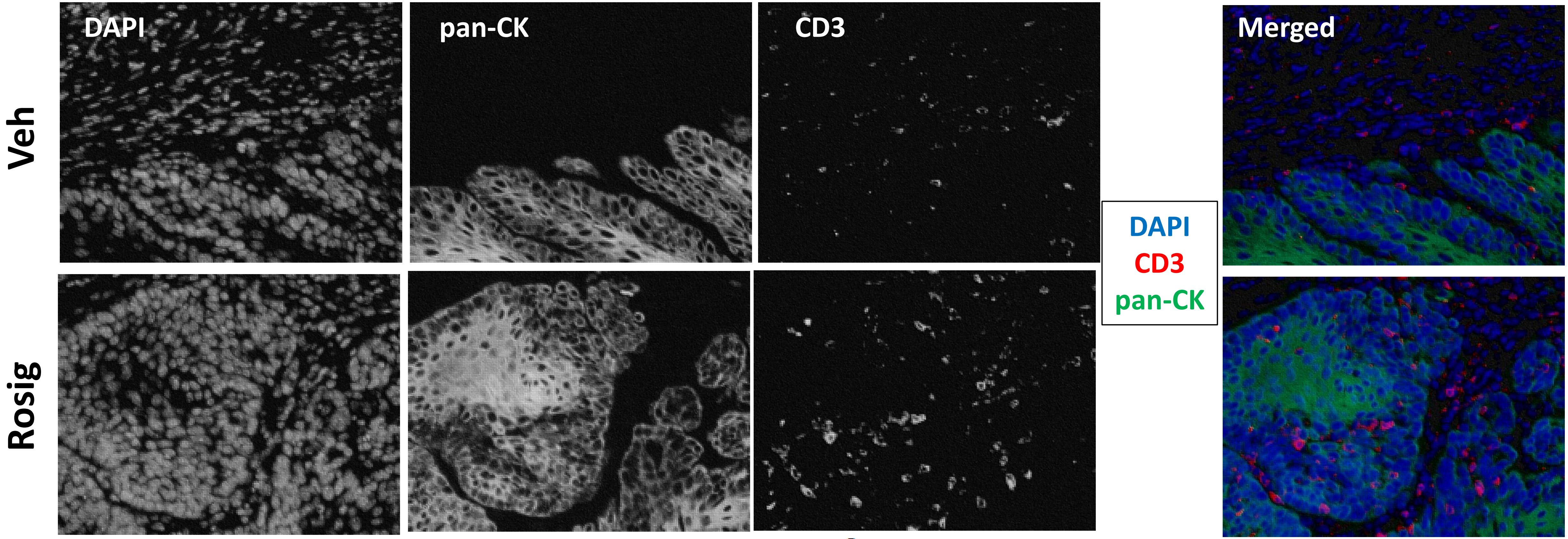

Figure S2: Immunofluorescent staining shows an increase in tumor-infiltrating $CD3^+$ cells in tumors that survived for 59 days. Photomicrographs (400x) are shown above of a typical rosiglitazone (Rosig) and vehicle (Veh) treated PDV tumor after 59 days. Individual panels for DAPI-stained nuclei, pan-CK+ tumor cells, and $CD3^+$ cells are shown, along with a merged image. An increase in $CD3^+$ cells is seen within the tumor which was labeled using pan-cytokeratin (pan-CK) antibodies. To the right are tissue cytometry plots showing the numbers of $CD3^+$ (Red or y-axis) and pan-CK^+ cells (Green or x-axis). $CD3^+$, pan-CK^+, double negative, and double positive cells are shown as a percentage of total DAPI- stained nuclei.

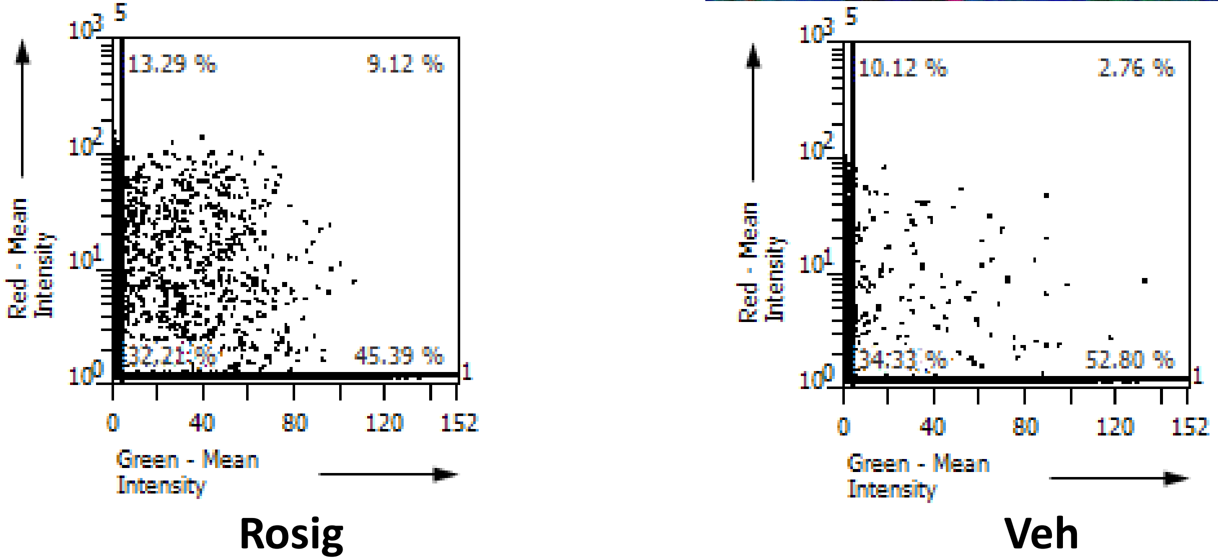

Figure S3: The ratio of total $CD3^+$ cells relative to myeloid cells within the tumor microenvironment is altered by rosiglitazone treatment. Total $CD3^+$ cells were determined for each tumor in figure 3 (Day 11) & figure 4 (Day 59). Similarly, the sum of all myeloid cell populations (expressing CD11b+, Gr-1+, and $CD11b^+Gr\text{-}1^+$) were also detected for each tumor. Rosiglitazone treatment promotes a time dependent increase in the ratio of $CD3^+$ to all myeloid cells (the sum of $CD11b^+ + Gr\text{-}1^+ + CD11b^+Gr\text{-}1^+$ cells). The shift to a CD3 predominant inflammatory response (Ratio > 1.0), was only seen at day 59, but not in early tumors at day 11. *, $p<0.05$; **, $p<0.01$; One-way ANOVA with Tukey's Multiple Comparison Test.

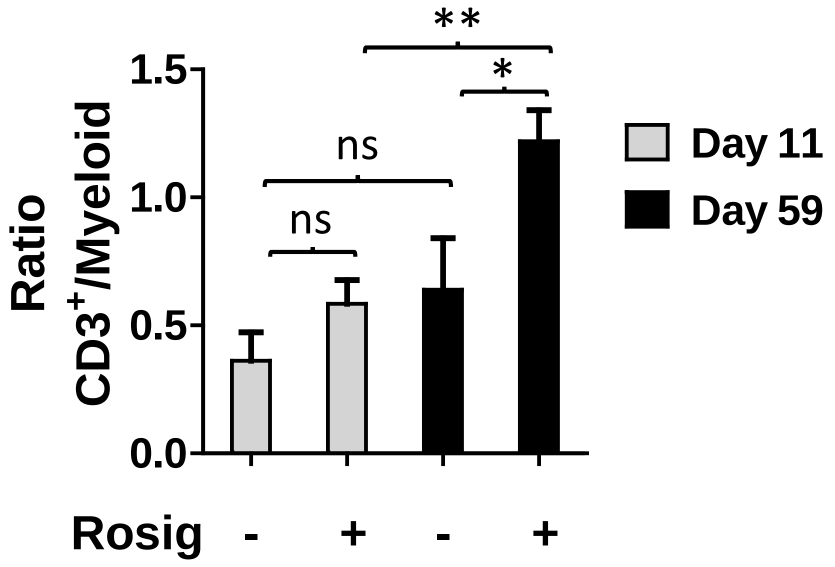

Supplement: Supplementary file 1 [file molecules-24-02192-s001.pdf]
